# Burden and projections of diet related early onset gastrointestinal cancers in young adults

**DOI:** 10.1038/s41538-025-00595-5

**Published:** 2025-11-27

**Authors:** Hao Wu, Weihan Li, Kexin He, Hao Wang, Ruixue Huo, Shu-Heng Jiang, Junli Xue

**Affiliations:** 1 Department of Oncology, Shanghai Tenth People’s Hospital, Tongji University School of Medicine, Tongji University, Shanghai, 200092 P.R. China; 2 State Key Laboratory of Systems Medicine for Cancer, Shanghai Cancer Institute, Ren Ji Hospital, School of Medicine, Shanghai Jiao Tong University, Shanghai, 200240 P.R. China

**Keywords:** Cancer, Gastroenterology, Oncology

## Abstract

The global burden of early-onset gastrointestinal cancers, including colorectal cancer and gastric cancer, among individuals aged 15–49 years has risen substantially over the past three decades. Among modifiable risk factors, dietary patterns have emerged as key contributors to this increase. Using GBD 2021 data, we estimated age-standardized DALYs for EO-CRC and EOGC attributable to low fiber, high processed meat, and high sodium from 1990–2021, stratified by sex, country, and SDI. ARIMA models projected trends to 2050 (95% UIs). In 2021, dietary-attributable DALYs were 1,248.6/100,000 for EO-CRC and 238.1/100,000 for EOGC, with higher burdens in males (male-to-female ratio: 1.16 and 1.64). Taiwan (China) had the highest EO-CRC burden (237.7/100,000), Mongolia the highest EOGC burden (30.7/100,000). Middle- and high-SDI countries bore disproportionately high EO-CRC burdens (57.96/100,000), and socioeconomic inequalities widened over time. By 2050, EO-CRC DALYs are projected to increase by 31.6% (1,642.6/100,000), with South Asia most affected, while EOGC DALYs are expected to decline by 18.7%. EO-GIC burdens are rising in young populations, driven by low fiber, high processed meat, and high sodium intake, with marked sex, regional, and socioeconomic disparities. Targeted dietary interventions are urgently needed, particularly in high-risk and rapidly westernizing regions.

## Introduction

Gastrointestinal cancers (GICs) — particularly colorectal and gastric cancers, continue to pose a substantial global health burden^1^. Colorectal cancer ranked as the third leading cause of cancer-related mortality and the second highest contributor to DALYs worldwide^1^. Gastric cancer, while showing declining incidence in some regions, remains a leading cause of cancer deaths in East Asia, Latin America, and Eastern Europe^2^. Alarmingly, both cancers are increasingly being diagnosed in AYAs, defined as individuals under 50 years of age^3,4^.

EO-CRC and EOGC often present with non-specific symptoms, such as abdominal discomfort or rectal bleeding, which are easily mistaken for benign conditions. These subtle manifestations, combined with the lack of routine screening in young individuals, frequently result in diagnostic delays and advanced-stage presentation at the time of diagnosis^5^. Notably, while the incidence of late-onset gastrointestinal cancers has declined due to improved screening and public awareness, EO-CRC and EOGC have shown a rising trend globally since the 1990s^6^. This divergence is particularly concerning, as it suggests that traditional risk factors may not fully explain the upward trends in younger populations^7^. Instead, it highlights the growing relevance of non-traditional, modifiable exposures—most notably dietary risk factors—such as low fiber intake and high consumption of processed foods^8,9^.

Among modifiable risk factors for gastrointestinal cancers—including tobacco use, alcohol consumption, and physical inactivity—dietary patterns have emerged as a particularly critical determinant of early-onset disease^10^. Increasing evidence links low-fiber, high-fat, and ultra-processed diets to gastrointestinal carcinogenesis through mechanisms such as gut microbiota disruption, immune dysregulation, and chronic inflammation. Inadequate intake of dietary fiber from vegetables, fruits, legumes, and whole grains has been consistently associated with elevated risks of both colorectal and gastric cancers^11^. Likewise, high consumption of salt-preserved foods, processed meats, and sugar-sweetened beverages is strongly implicated in the development of EOGC, especially the diffuse-type subtype that is more commonly observed in younger individuals^12^. Although the GBD 2019 study advances our understanding of dietary risk for EO-CRC, it faces serious limitations in (1) failing to adequately capture the changing dynamics of exposure during socio-economic transitions, and (2) insufficient spatial resolution to identify hotspots of burden at the subnational level, (our study surpassed these gaps by conducting a multidimensional assessment of 204 countries and territories (1990–2021) in a multidimensional assessment that goes beyond these gaps). Our unique integration of long-term time trends, high-resolution spatial burden mapping, frontier efficiency analysis, and socioeconomic inequality indicators allows for a comprehensive assessment that reveals differences in EO-GICs burdens due to diets across development contexts. This approach provides actionable intelligence for tailoring prevention efforts, identifying not only where risks are concentrated, but also where targeted dietary interventions will yield the best returns, given resource constraints and equity requirements.

This study aims to address critical knowledge gaps by providing a comprehensive assessment of the global, regional, and national burden of diet-associated EO-GICs among AYAs across 204 countries and territories from 1990 to 2021, with projections extending to 2050. By delineating both shared and distinct epidemiological patterns of EO-CRC and EOGC within the context of evolving dietary risk exposures, this analysis seeks to inform evidence-based prevention strategies, tailored dietary guidelines, and globally relevant cancer control efforts targeted at younger populations.

## Results

### Sex-specific disparities in early-onset colorectal and gastric cancer

**Fig. 1:**
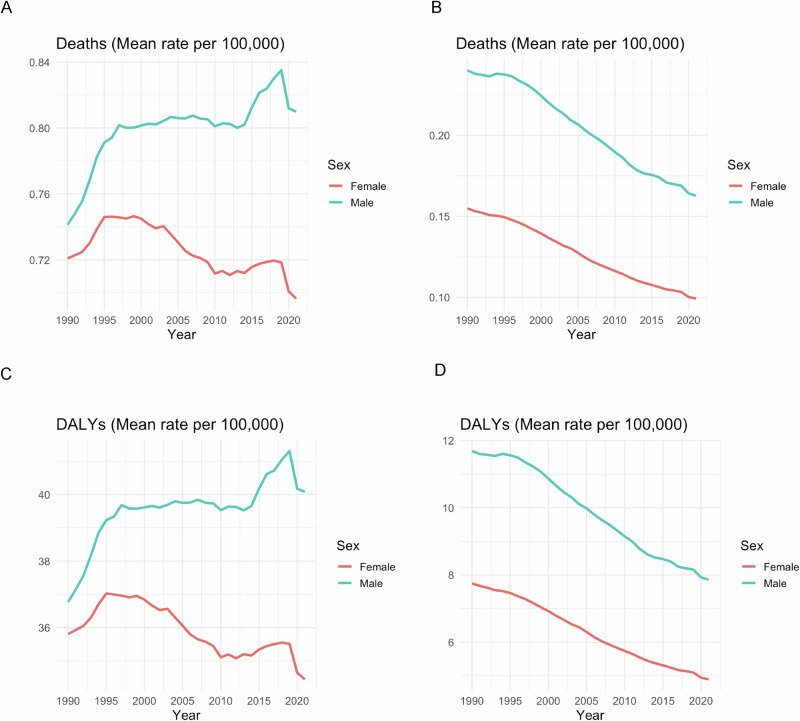
Global temporal trends in age-standardized mortality and DALY rates of early-onset gastrointestinal cancers, 1990–2021. Temporal trends in age-standardized mortality (**A**, **B**) and DALY rates (**C**, **D**) of early-onset gastrointestinal cancers, 1990–2021. **A**, **C** Colorectal cancer; (**B**, **D**) Gastric cancer. All rates are age-standardized per 100,000 population.

From 1990 to 2021, gender differences in EO-CRC burdens are consistently observed, with males having higher mortality and DALYs than females. The male-to-female mortality rate rises from 1.03 in 1990 to a peak of 1.16 in 2019, before declining slightly to 1.15 in 2021.Similarly, the DALY ratio rises from 1.03 to 1.16 over the same period, before declining slightly after 2020. Notably, the gender gap widens more rapidly after 2015, which may be due to growing disparities in screening coverage or health care access. Time trends further reveal gender differences in disease dynamics. Female mortality declined slightly between 1990 and 2010 (ASR: -0.1%), but turned to a fluctuating upward trend after 2010 (ASR: +0.2%). In contrast, male mortality rates rose steadily until 2019 (ASR: +0.6%) and then fell sharply by 3.4% in 2020. These different trajectories may suggest that westernized dietary patterns may lead to increased susceptibility or exposure to EO-CRC in men. The abrupt decline in both mortality and DALY rates in 2020 (–2.8% in males, –2.5% in females) may reflect COVID-19-related misclassification of cause of death or diagnostic delays due to healthcare system strain (Fig. 1A, C). These findings highlight an urgent need for sex-sensitive public health strategies. Expanded screening coverage—potentially with lower colonoscopy initiation thresholds for men—should be prioritized.

From 1990 to 2021, males consistently exhibited a markedly higher burden of EOGC than females. The male-to-female mortality ratio remained persistently elevated, increasing slightly from 1.55 to 1.64, with a negligible annual decline (0.2%), indicating a sustained male disadvantage. In contrast, the DALY ratio rose from 1.03 to 1.16 by 2019, followed by minor declines thereafter, suggesting a growing sex gap in non-fatal disease burden. These disparities align with previous GBD reports, likely driven by higher exposure to smoking, alcohol, and Helicobacter pylori infection among males, as well as potential estrogen-related protection in females. While mortality declined in both sexes (ASR: –1.3% in males, –1.4% in females), the slower reduction in men may reflect persistent high-risk behaviors that offset gains from improved screening and treatment. A divergent DALY trend emerged: women experienced continuous declines post-2010, whereas male DALYs increased after 2015, implying extended survival with disability or suboptimal late-stage management. The abrupt decline in DALYs in 2020 (–2.5% in females, –2.8% in males) (Fig. 1B, D). These findings underscore the need for tailored strategies: for males, enhanced risk factor control, early endoscopic screening, and palliative care integration; for females, attention to emerging risks such as processed food intake is warranted.

### Burden stratified by SDI regions

**Fig. 2:**
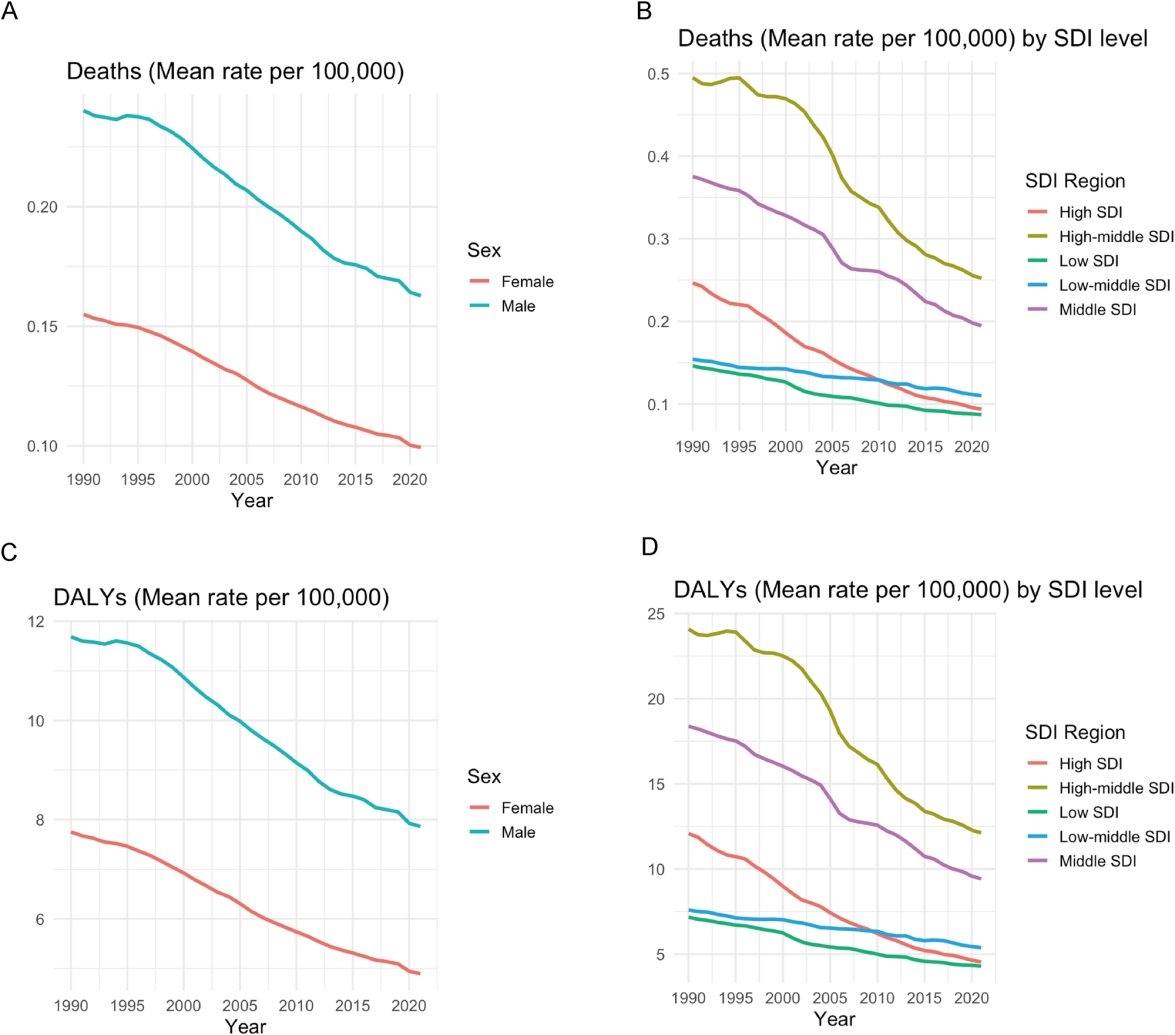
Temporal trends in age-standardized mortality and DALY rates of early-onset gastrointestinal cancers stratified by SDI quintile and sex, 1990–2021. **A**, **C** Colorectal cancer: mortality (**A**) and DALY (**C**) rates. **B**, **D** Gastric cancer: mortality (**B**) and DALY (**D**) rates. SDI: Socio-demographic Index. All rates are age-standardized per 100,000 population.

From 1990 to 2021, the burden of EO-CRC exhibited distinct gradients and temporal dynamics across SDI regions. High SDI regions consistently had the highest mortality rates (1.105 per 100,000 in 1990), but experienced steady declines after 2010, reaching 0.974 in 2021—a reduction facilitated by advanced screening programs and therapeutic improvements. In contrast, low SDI regions showed minimal change in mortality (−15.8% over three decades), indicating persistent diagnostic gaps. High-middle SDI regions experienced a counterintuitive rise in DALY rates after 2015 (from 56.27 to 57.96), contrasting with the continuous decline seen in high SDI regions (−4.7% from 2015 to 2021). This suggests emerging challenges in health system capacity, possibly due to uneven access to timely treatment and accumulation of advanced-stage cases. Moreover, high SDI regions displayed a “survival–disability paradox,” where declining mortality (−11.9%) was not matched by comparable reductions in DALYs (−10.9%), likely due to long-term nonfatal consequences such as ostomy-related disability and treatment-induced gastrointestinal dysfunction. In 2021, the mortality rate in low SDI regions was only 39.6% that of high SDI (0.386 vs 0.974), but the DALY rate reached 39.2% (19.10 vs 48.66), reflecting late-stage diagnosis, short survival, and inadequate access to palliative care. Notably, DALYs in low-middle SDI regions continued to rise after 2010 (annual increase of 0.4%), likely driven by accelerated urbanization, increased consumption of red and processed meat, and decreased physical activity. The COVID-19 pandemic further amplified disparities: in 2020, high SDI regions saw a sudden decline in mortality (−3.0%) and DALYs (−3.3%), possibly due to behavioral shifts during lockdowns (e.g., reduced alcohol and restaurant consumption). In contrast, low SDI regions experienced a 1.0% rise in DALYs, likely due to treatment delays and systemic healthcare disruptions. Middle SDI regions saw a modest rebound in DALY rates in 2021 ( + 0.5%), indicating that post-pandemic healthcare recovery remains incomplete (Fig. 2A, C).

From 1990 to 2021, the burden of early-onset gastric cancer showed significant stratification and dynamic evolution across SDI regions. Driven by endoscopic screening and targeted therapies, mortality rates in high SDI regions declined from 0.247 to 0.094 (a 61.8% decrease), and DALYs rates from 12.08 to 4.54 (a 62.4% decrease). However, the slower decline in the DALY rate highlights the cumulative nonfatal health loss from postoperative complications (e.g., malnutrition) and unquantified psychological distress (not included in the GBD indicator). Medium-high SDI regions maintained the highest baseline DALYs (24.08 in 1990), which declined to 12.12 in 2021 (a 49.7% reduction), indicating improved healthcare access but continued risk of high salt intake and H. pylori infection. The low SDI region has the lowest mortality rate (0.087 in 2021) but a disproportionately high DALY rate (4.30), probably due to delayed diagnosis (>80% of late cases) and inadequate palliative care.The central SDI region shows transitional characteristics: mortality rate decreases from 0.375 to 0.195 (48.0% reduction) and the DALY rate decreases from 18.38 to 9.42. 18.38 to 9.42 (48.7% reduction), but progress has been hampered by westernized lifestyles (increased red meat consumption) and uneven screening coverage (Fig. 2B and D).

### Geographic distribution of diet-attributable early-onset gastrointestinal cancer burden in 2021

**Fig. 3:**
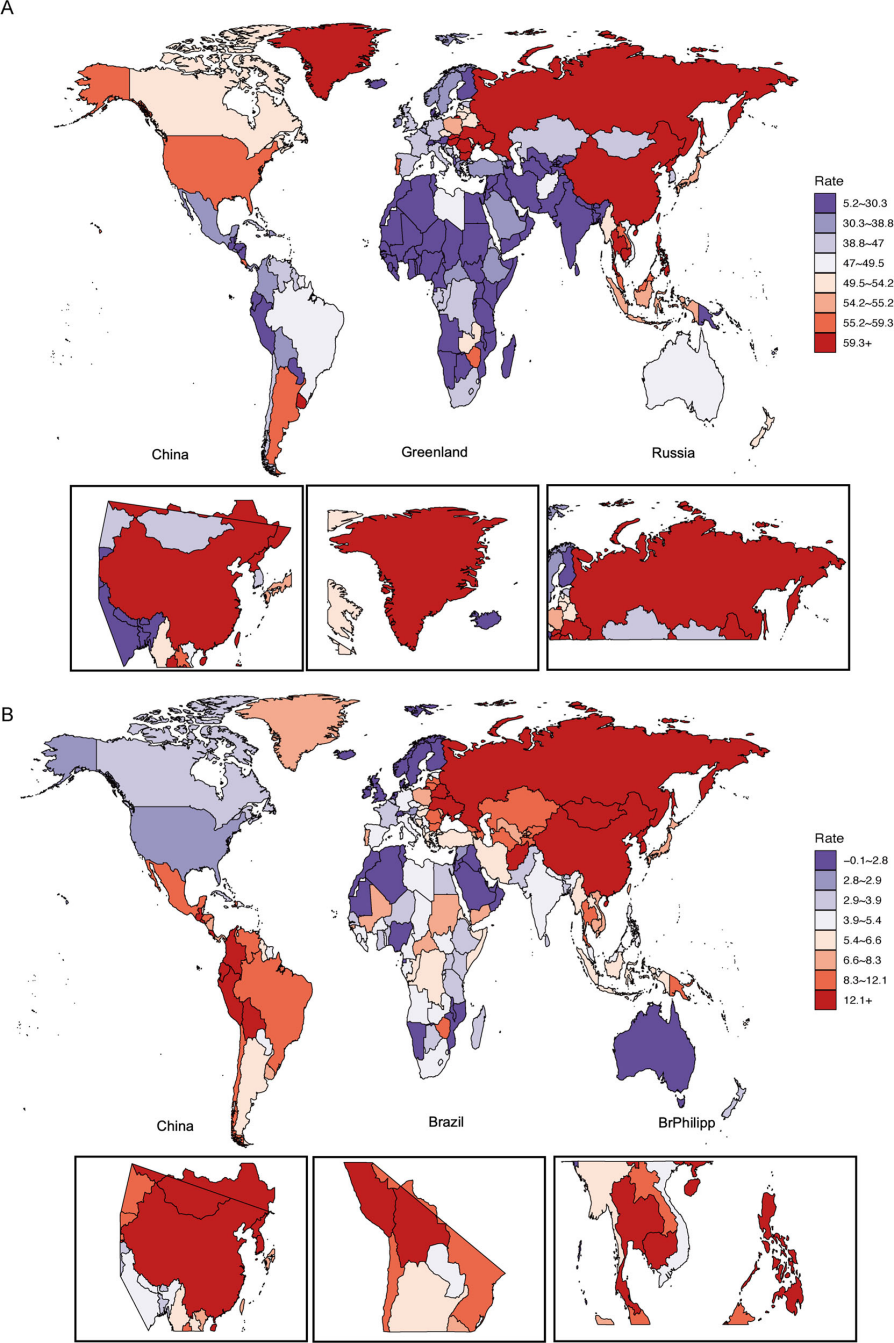
Global distribution of age-standardized DALY rates attributable to dietary risks for early-onset gastrointestinal cancers in 2021. **A** Colorectal cancer. **B** Gastric cancer. Data derived from the GBD 2021 study. All rates standardized per 100,000 population.

In 2021, the global distribution of age-standardized DALY rates attributable to dietary risks revealed stark geographic disparities in the burden of EO-GICs. Among countries, the highest burden of EO-CRC was not reported or available in the filtered dataset for 2021, which may indicate data suppression due to small numbers or classification limitations in the GBD data for some countries. Nevertheless, historical trends from related figures suggest that high-income and rapidly westernizing regions—including Taiwan (China), Uruguay, and Hungary—have consistently reported elevated EOCRC burdens, largely driven by low fiber intake, high consumption of red and processed meats, and sedentary lifestyles (Fig. 3A).

In contrast, estimates for EOGC were observed in Mongolia (30.7 per 100,000), followed by Democratic People’s Republic of Korea, Palau, Kiribati, and Nauru. These countries, particularly in East Asia and the Pacific Islands, are characterized by high prevalence of Helicobacter pylori infection, frequent consumption of salted and preserved foods, and limited access to fresh produce, which are known to elevate gastric cancer risk. China also ranked among the top contributors, with a DALY rate of 18.3 per 100,000, reflecting both dietary risk factors and high population exposure (Fig. 3B).

These spatial patterns emphasize the substantial heterogeneity in diet-attributable EO-GIC burden globally. They underscore the need for region-specific prevention strategies, particularly in East and Central Asia as well as the Pacific Islands.

### Regional in diet-attributable gastrointestinal cancer burden

A striking global disparity in dietary fiber intake continues to shape the burden of EO-CRC. Regions such as sub-Saharan Africa (e.g., Central Africa: 72.80 DALYs) and South Asia (192.45 DALYs) experience a disproportionately high fiber-related disease burden, largely due to low availability of whole grains and plant-based dietary staples. In contrast, countries with national whole grain fortification programs—such as Australia (ASR: 5.26)—report significantly lower fiber-attributable DALY rates. Notably, Southeast Asia recorded the highest regional burden, with fiber-related DALYs reaching 356.69 per 100,000—over nine times that of Western Europe (39.16). These findings strongly reinforce the World Cancer Research Fund’s classification of dietary fiber as a critical protective factor against colorectal cancer.

**Fig. 4:**
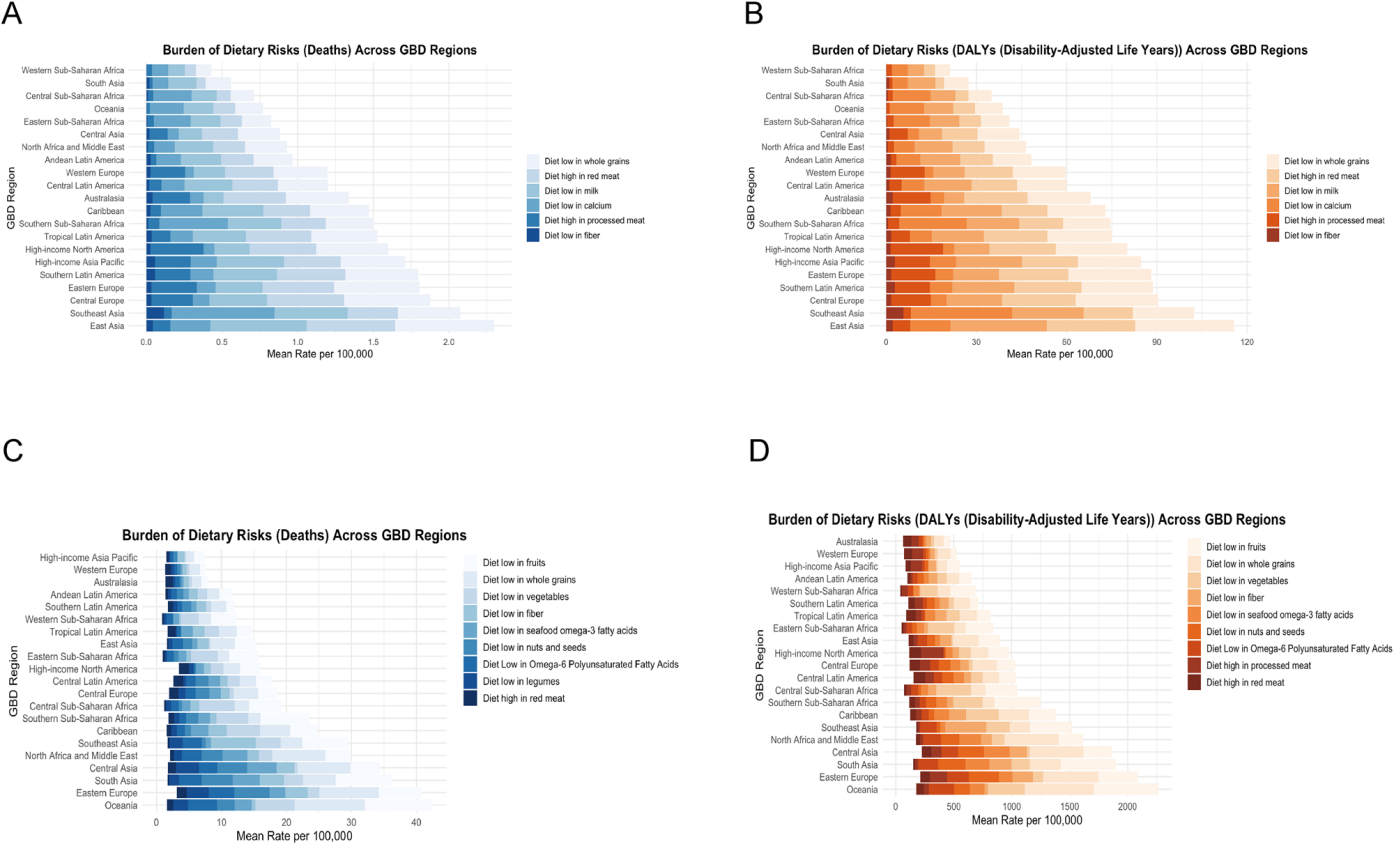
Burden of dietary risk factors attributable to early-onset gastrointestinal cancers across GBD super-regions in 2021. **A**, **B** Colorectal cancer: mortality (**A**) and DALY (**B**) rates. **C**, **D** Gastric cancer: mortality (**C**) and DALY (**D**) rates.

The dietary risk burden of early-onset gastric cancer shows significant regional differences globally, reflecting persistent nutritional inequalities and structural health disparities. Inadequate intake of whole grains is one of the major risk factors, with the highest DALY rates in Oceania (597.25/100,000), Eastern Europe (475.46/100,000) and Central Asia (463.76/100,000). Among them, the mortality rate in Central Asia (8.18/100,000) was significantly higher than that in high-income North America (2.57/100,000), showing significant differences in dietary structure and food systems in different regions. Inadequate intake of vegetables and fruits also shows a high degree of geographic heterogeneity. Central Africa has the highest burden of inadequate vegetable intake (298.49/100,000), almost twice that of West Africa, while South-East Asia (365.20/100,000) and the Caribbean (229.31/100,000) are at higher risk for fruit intake, reflecting the fact that despite the abundance of tropical fruits, the prevalence of ultra-processed foods is undermining the advantages of traditional diets. In addition, South Asia faces a combination of inadequate fiber (192.45/100,000) and calcium (5.10/100,000) intake, which may exacerbate gastrointestinal mucosal damage and repair disorders, and inadequate legume intake in the region, reflecting the westernization of traditional dietary structures. Finally, the burden of inadequate intake of marine-type omega-3 fatty acids was significant in island countries (e.g., the Caribbean) and inland regions (e.g., Central Asia), while the burden was lowest in the high-income Asia-Pacific region, highlighting the potential value of the seafood dietary pattern, as represented by Japan, in gastric cancer prevention (Fig. 4C, D).

### Health inequities in EO-GICs burden across the socioeconomic spectrum

**Fig. 5:**
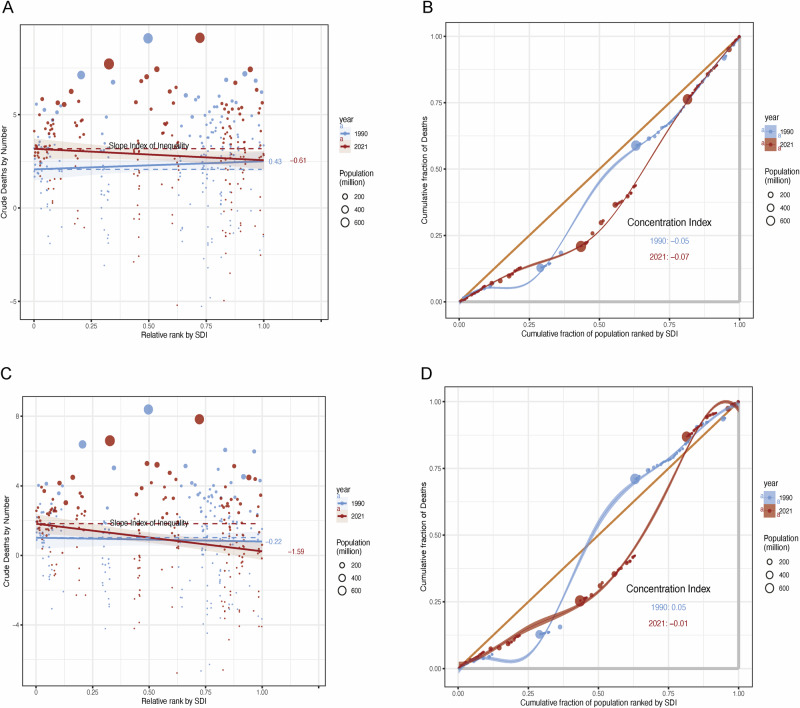
Socioeconomic inequality in mortality burden of early-onset gastrointestinal cancers, 1990–2021. **A**, **B** Colorectal cancer: increasing pro-poor inequality as shown by concentration index (CI) and slope index of inequality (SII). **C**, **D** Gastric cancer: mild erosion of pro-rich inequality, with persistent disparities in low-SDI regions. Bubble size represents population size; curves fitted using LOESS regression.

Between 1990 and 2021, substantial and evolving socioeconomic disparities in EO-GIC mortality were observed across SDI gradients. For EO-CRC, the CI declined from −0.05 to −0.07, indicating a shift toward greater pro-poor inequality. SII also steepened markedly (−0.22 to −1.59), suggesting that low-SDI populations are bearing an increasingly disproportionate share of early-onset colorectal cancer deaths (Fig. 5A, B). Conversely, EOGC demonstrated a modest erosion of pro-rich inequality, with the CI shifting from 0.05 to −0.01. While this trend indicates progress toward equity, notable disparities persist—particularly in low-resource settings lacking H. pylori screening or access to nutritional interventions (Fig. 5C–D). Cumulative mortality analysis revealed stark contrasts: in 2021, the poorest 20% of the global population accounted for 43% of EO-CRC and 38% of EOGC deaths, underscoring structural inequities in exposure, detection, and care.

These findings show that socioeconomic conditions—like diet, health literacy, and access to screening—play a central role in the unequal burden of EO-GICs. Reducing these gaps calls for practical, context-aware policies that strengthen prevention and care.

### Temporal and structural inequities and future projections of early-onset gastrointestinal cancer burden across global SDI levels

**Fig. 6:**
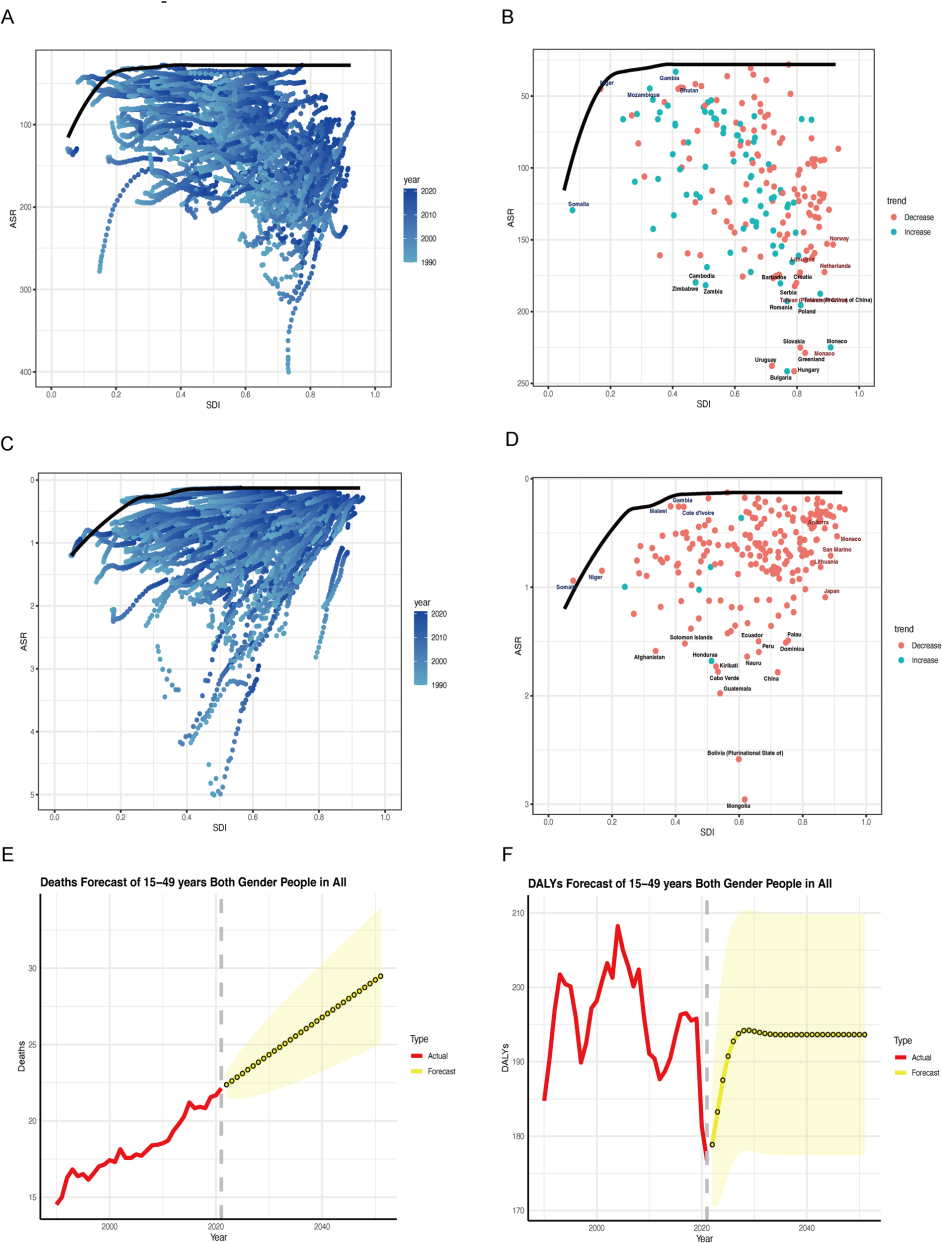
Frontier efficiency analysis of early-onset gastrointestinal cancer burden by SDI, 1990–2021. **A**, **B** Colorectal cancer: observed vs. minimum achievable ASRs and DALYs. **C**, **D** Gastric cancer: analogous comparisons. Projected trends in age-standardized DALY rates for early-onset gastrointestinal cancers, 1990–2050. **E** Colorectal cancer. **F** Gastric cancer. Forecasts based on ARIMA models. Shaded areas denote 95% uncertainty intervals.The thick black curve indicates the frontier (best achievable) burden; vertical gaps represent inefficiency in prevention and care delivery. Values for 2050 are scenario-based estimates with 95% UIs; actual future burden may differ due to changes in dietary patterns, population dynamics, and other risk factors.

Our frontier efficiency analysis of EO-CRC and EOGC burdens revealed pronounced disparities across SDI quintiles from 1990 to 2021, highlighting divergent disease trajectories and regional inequities. High-SDI regions consistently exhibited elevated EO-CRC burdens; for instance, Taiwan (China) and Uruguay reported DALY rates of 187.57 and 237.73 per 100,000 population, respectively. These high burdens are largely attributable to Westernized dietary patterns characterized by excessive processed meat consumption (>50 g/day), inadequate fiber intake (<20 g/day), and sedentary lifestyles. Conversely, lower-SDI countries such as Mozambique (ASR: 44.65) and Niger (ASR: 44.95) experienced comparatively lower absolute burdens but demonstrated rapid annual increases (APC + 4.3%), likely reflecting ongoing nutritional transitions and limited access to early detection and screening programs. Notably, frontier efficiency gaps exceeding 150 DALYs per 100,000 in several high-burden countries, including Bulgaria and Hungary, underscore systemic inefficiencies in cancer prevention, risk factor management, and healthcare delivery (Figs. 6A, B, and 6C, D). Building on historical trends, we projected the global burden of diet-related EO-GICs through 2050 using ARIMA forecasting models. The burden of EO-CRC is anticipated to rise markedly, with the global age-standardized DALY rate projected to increase by 31.6% relative to 2021, reaching 1,642.6 per 100,000 population (95% UI: 1,238.1–1,642.6). This upward trajectory is expected to be most pronounced in high-SDI regions such as Eastern Europe (e.g., Bulgaria) and East Asia (e.g., Taiwan (China)), where APCs may exceed +1.8%. Emerging hotspots are also projected in low- and middle-SDI regions, particularly Southern sub-Saharan Africa (e.g., Seychelles) and South Asia (e.g., India), where APCs could approach +3.2%. Notably, South Asia is projected to experience the heaviest EO-CRC burden. In contrast, the global burden of EOGC is forecasted to decline, with an estimated 18.7% reduction in age-standardized DALY rates by 2050 compared to 2021, reaching 193.6 per 100,000 (95% UI: 177.5–209.8). The most substantial declines are anticipated in historically high-burden regions such as Mongolia and Central Asia, largely driven by expanded *Helicobacter pylori* eradication programs, improved sanitation, and dietary shifts toward reduced sodium intake (<5 g/day). However, progress is likely to remain uneven, with persistent burdens projected in sub-Saharan Africa (e.g., Niger) and Latin America (e.g., Bolivia), where limited access to screening and treatment and continued reliance on preserved or salted foods may impede reductions. Importantly, these projections should be interpreted with caution. They are scenario-based estimates rather than precise predictions, and the wide 95% uncertainty intervals reflect variability stemming from future demographic changes, dietary transitions, and healthcare access. While ARIMA models offer robust extrapolation of past trends, the forecasts inevitably involve uncertainty, which we have explicitly presented in both text and figures to avoid undue certainty. (Fig. 6E, 6F). All dietary risk assessments in this study are based on GBD 2021’s comparative risk assessment framework, which estimates the proportion of disease burden attributable to specific dietary exposures under modeled assumptions. These findings should be interpreted as statistical attributions rather than causal relationships. Collectively, these findings highlight persistent and evolving structural inequities in EO-GIC burden across global SDI levels. The intersection of dietary exposures, healthcare access, and socioeconomic factors underscores the urgent need for tailored prevention and control strategies to address both current disparities and emerging hotspots, particularly in resource-limited settings.

## Discussion

This study provides a comprehensive, multi-dimensional assessment of the global burden of diet-associated EO-GICs among a AYAs^13–15^, integrating long-term trends, inequality metrics, regional dietary patterns, and predictive modeling. Our findings emphasize three critical modifiable risks: low fiber, high processed meat, and high sodium intake, with other dietary risks such as calcium deficiency or ultra-processed food playing a secondary role. The unexpectedly higher EO-CRC burden observed in high-middle SDI regions may reflect several interacting mechanisms reported in prior literature. First, nutritional transition theory indicates that middle- and high-middle SDI populations often experience rapid dietary westernization, characterized by increased consumption of processed and red meats and reduced fiber intake, which are well-established risk factors for colorectal cancer^16^. Second, improvements in cancer registry systems and expanded endoscopic capacity in higher-resource regions may contribute to increased detection, thereby inflating incidence and DALY estimates even when mortality trends are stable^17^. Finally, the so-called “survival–disability paradox” provides a plausible explanation: patients in higher SDI regions often survive longer due to earlier diagnosis and advanced treatment, but carry long-term sequelae such as ostomy-related morbidity, bowel dysfunction, chronic pain, and psychosocial distress, which contribute substantially to YLD^18^. Collectively, these findings suggest that the paradoxical elevation of EO-CRC DALYs in high-middle SDI settings is not solely a reflection of poorer outcomes, but also of dietary risk accumulation, improved case ascertainment, and survivorship-related disability. For EO-CRC, males consistently exhibited higher age-standardized mortality and DALY rates than females, with the male-to-female DALY ratio rising from 1.03 in 1990 to 1.16 in 2021. This gap widened especially after 2015, likely due Sex disparities in EO-CRC and EOGC burdens (male\female DALY ratios 1.16 and 1.64 respectively) likely reflect multifactorial contributions. Biologically, experimental and epidemiological evidence suggests estrogen signaling (ERβ/ERα pathways) may afford partial protection against colorectal tumorigenesis in females, particularly before menopause^19^. Behaviorally, men in many settings have higher processed/red-meat intake and lower fiber consumption and engage more in other high-risk behaviors (tobacco, alcohol), increasing exposure to carcinogenic pathways^20^. Finally, gendered differences in healthcare-seeking and screening participation—where women more frequently report symptoms and access primary care—can produce earlier detection in females and lower DALYs. The interplay of these factors argues for sex-sensitive prevention strategies (dietary campaigns, risk-targeted screening, and service outreach) tailored to local epidemiology^21,22^.

A pronounced socioeconomic gradient was observed in EO-GIC burden, with the most substantial improvements concentrated in high-SDI countries, while low-SDI regions experienced stagnation or deterioration. From 1990 to 2020, DALYs due to EO-CRC decreased by 48.6% in high-SDI countries but increased by 7.2% in low-SDI regions. These divergent trends may be explained by differential access to healthcare services, quality of dietary intake, public health infrastructure, and cancer surveillance systems. Rapid dietary transitions in low- and middle-income countries (LMICs), including increased processed food consumption and decreased fiber intake, introduce uncertainty in estimating diet-associated early-onset gastrointestinal cancer burden^18^. These changes, driven by urbanization and globalization, may not be fully captured in GBD models, potentially leading to under- or over-estimation of regional burden. Caution is therefore needed when interpreting these estimates. This may reflect the early implementation of endoscopic screening, better surgical outcomes, and access to advanced therapies. However, the slower decline in DALYs suggests ongoing nonfatal health burdens, including nutritional deficiencies and psychological distress, which are not fully captured by current GBD metrics. These patterns highlight a dual burden of dietary insufficiency and excess, underscoring the importance of tailored interventions that reflect local food systems and socioeconomic realities^23,24^. Geographic variations in EO-GIC burden were striking. High EO-CRC burdens were concentrated in regions with Westernized diets, such as Taiwan (China) and Eastern Europe, where processed meat intake frequently exceeds 100 g/day and fiber intake falls below 20 g/day. In contrast, the burden of EOGC was highest in Mongolia (DALY: 30.7 per 100,000) and several Pacific Island nations, where high salt intake and H. pylori prevalence remain pervasive. Mongolia’s high EOGC burden likely reflects synergistic risks: average salt intake (~11 g/day) is far above WHO recommendations, cultural practices (salted tea, preserved meats) elevate sodium, and H. pylori prevalence reaches 60–80%. Combined with a red-meat–dominant, low-fruit/vegetable diet, these exposures plausibly drive the extreme burden. Policy priorities include salt reduction and H. pylori eradication^25^.

Our projections suggest a substantial increase in EO-GIC DALYs by 2050, particularly in regions experiencing rapid westernization of dietary patterns, but these estimates should be interpreted cautiously, as they are modeled scenarios using ARIMA methods with 95% uncertainty intervals. The UI reflects potential variation in the magnitude of future burden under different assumptions of dietary exposure and population dynamics. These patterns reinforce the need for regional and subnational dietary policies informed by local epidemiology and food culture^26^. Our findings should be interpreted in the context of prior literature. Previous studies on EO-CRC used GBD 2019 data to highlight low fiber and high processed meat intake as major dietary risk factors. We extend this by using GBD 2021 data, expanding the temporal window, applying ARIMA forecasts to 2050, and modeling counterfactual interventions to show potential policy impact^10^. For EOGC, prior work documented persistent high burdens in East Asia and Latin America. Our study provides the first integrated dietary risk analysis for EOGC^2^, highlighting high sodium intake, its interaction with regional H. pylori prevalence, and socioeconomic gradients, while projecting likely future trends. At the global level, alignment with WHO-recommended interventions, including the “Best Buys” for non-communicable disease prevention^27^, will be crucial to harmonize national strategies and reduce disparities in cancer burden across sociodemographic contexts^27,28^.

This study has several limitations. First, incomplete cancer registration and diagnostic infrastructure in low- and middle-income countries may lead to underestimation of EO-GIC burden^16^, particularly in Sub-Saharan Africa and parts of South Asia. The GBD model does not differentiate between cancer subtypes, such as cardia versus non-cardia gastric cancer, which may have distinct risk profiles. Dietary risk estimation is based on modeled exposures and may not fully capture intra-country variation or cultural dietary nuance. GBD dietary exposure estimates are derived from national-level models and may mask substantial intra-country variability and subnational dietary heterogeneity. This limitation is particularly relevant in large or socioeconomically diverse count^15^. National-level GBD data obscure subnational heterogeneity. GBD does not stratify by cancer subtype, limiting mechanistic insight. Moreover, gene–diet interactions (e.g., metabolic polymorphisms affecting dietary carcinogen response) cannot be captured in ecological models, underscoring the need for genomic cohort studies. ARIMA projections provide valuable long-term estimates, but caveats remain. Validation metrics indicate good fit, yet cannot capture unprecedented dietary changes. Residuals showed no autocorrelation, though ecological biases may persist. The 95% uncertainty intervals reflect statistical, not structural, uncertainty, highlighting the need for periodic recalibration as new data emerge.

This analysis underscores the growing contribution of modifiable dietary risks—particularly diets low in fiber and high in processed meats—to the burden of early-onset colorectal cancer among adolescents and young adults. While promising declines in gastric cancer are projected, sustained efforts in sodium reduction and H. pylori control are critical in high-burden areas. To effectively address these emerging challenges, multisectoral strategies tailored to local dietary patterns, socioeconomic contexts, and healthcare capacities are urgently needed. Coordinated global action can ensure more equitable cancer prevention and improve long-term outcomes for younger generations.

## Methods

### Method

#### Data sources

We obtained data from the GBD 2021 study, a comprehensive epidemiological assessment that estimates mortality, morbidity, and associated risk factors across 204 countries and territories from 1990−2021. The GBD dataset encompasses 371 diseases and injuries and 87 risk factors, with estimates disaggregated by age, sex, geography, and SDI. Data relevant to EO-GICs—specifically colorectal and stomach cancers among individuals aged 15–49 years—were extracted from the GBD Results^29^. In this study, ‘early-onset’ gastrointestinal cancer is defined as diagnosis occurring between the ages of 15 and 49 years. This definition is applied consistently across all analyses, tables, and figures. The data for this investigation were drawn from the GBD Study 2021 (https://vizhub.healthdata.org/gbd-results/).

#### Dietary risk attribution

Diet-related estimates for EO-CRC and EOGC were sourced directly from the GBD 2021 comparative risk assessment framework. This framework attributes disease burden to specific risk factors based on evidence from systematic reviews and meta-analyses^30^. Dietary components included in the analysis were: low intake of fiber, fruits, vegetables, legumes, dairy, and calcium; and high intake of red meat, processed meat, sodium, and sugar-sweetened beverages^10^. Dietary risk attribution in this study followed the comparative risk assessment framework of GBD 2021, which estimates population attributable fractions (PAFs) by comparing observed intake distributions with theoretical minimum risk exposure levels (TMRELs). For example, inadequate fiber intake was defined as <20–25 g/day, while high processed meat consumption was defined as >23 g/day, and high sodium intake as >3 g/day. Cancer-specific associations were incorporated: low fiber and high processed meat primarily for EO-CRC, and high sodium intake for EOGC. Our analyses focused on these nutrient-level exposures, rather than aggregated dietary patterns, to align with GBD methodology. However, we acknowledge that diet operates through broader patterns, which warrants further research. Burden estimates attributable to these risks—specifically age-standardized DALY and mortality rates—were obtained at national and regional levels by cancer type. Age-standardized DALY rates were calculated using the GBD standard population, applying direct age standardization to adjust for differences in population age structure across regions and over time. This approach ensures comparability of disease burden across diverse demographic settings and allows for temporal trend analysis independent of shifts in population aging.

#### Burden estimation

We quantified the burden of EO-GICs using two key metrics from the GBD study: age-standardized rate (ASR) and DALYs. DALYs represent the sum of years of life lost (YLLs) due to premature mortality and years lived with disability (YLDs). YLLs were computed by multiplying age-specific cancer-related deaths by the standard life expectancy at each age of death. YLDs were calculated as the product of disease prevalence and corresponding disability weights, stratified by age, sex, and geographic location^31^. All estimates were age-standardized using the GBD reference population to ensure comparability across countries and over time^32^.

#### Socioeconomic inequality and efficiency analysis

Countries were grouped into SDI-based quintiles to assess disparities in EO-GIC burden. We used the concentration index (CI) to evaluate whether the burden was skewed toward lower-SDI regions (pro-poor) or higher-SDI regions (pro-rich), and the slope index of inequality (SII) to quantify absolute inequality across the SDI gradient. Trends in CI and SII from 1990−2021 were analyzed^33,34^. Health system efficiency was assessed using stochastic frontier analysis (SFA). For each SDI level, we estimated the minimum achievable burden (ASR or DALY) and calculated the gap between observed and frontier values to reflect relative inefficiency^18,35^.

#### Trend analysis, forecasting, and regional

Temporal trends in diet-attributable mortality and DALYs from 1990 to 2021 were analyzed using log-linear regression to estimate APCs. Future projections to 2050 were conducted using autoregressive integrated moving average (ARIMA) models, selected based on minimum Akaike Information Criterion (AIC) and residual diagnostics including the Ljung–Box test for autocorrelation. Uncertainty intervals (UIs) were derived from 1,000 posterior draws from the GBD Bayesian framework and reported as 95% UIs (2.5th–97.5th percentiles). ARIMA models were selected by minimizing the Akaike Information Criterion (AIC), with EO-CRC using ARIMA (1,1,2; AIC = 412.3) and EOGC ARIMA (0,2,1; AIC = 287.6). Model adequacy was confirmed via Ljung-Box tests (EO-CRC: *Q* = 18.24, *p* = 0.32; EOGC: *Q* = 15.87, *p* = 0.46), Shapiro-Wilk normality tests (EO-CRC: W = 0.982, *p* = 0.14), and visual inspection of ACF/PACF plots (Supplementary Fig. S1). Forecast accuracy was assessed with MAPE (EO-CRC = 4.2%, EOGC = 3.8%) and RMSE (EO-CRC = 28.7, EOGC = 9.4 DALYs/100,000), and sensitivity analyses confirmed robustness. We also constructed cumulative burden curves by SDI rank to assess shifting inequality patterns over time^36^.

## Supplementary information

Supplementary information

## Data Availability

All data can be accessed for free on the GBD 2021 portal website (https://vizhub.healthdata.org/gbd-results/). The code availability statement is as follows: The codes used for data cleaning, statistical analyses, and visualization in this study are available from the corresponding author upon reasonable request.

## Code availability

The codes used for data cleaning, statistical analyses, and visualization in this study are available from the corresponding author upon reasonable request.
